# A comparative study of psychological factors in Men who have Sex with Men (MSM) with and without HIV

**DOI:** 10.1192/j.eurpsy.2024.634

**Published:** 2024-08-27

**Authors:** K. E. Sgoura, A. Papadopoulou, V. Efstathiou

**Affiliations:** ^1^Psychology Department, National and Kapodistrian University of Athens; ^2^Second Department of Psychiatry, National and Kapodistrian University of Athens, “Attikon” University General Hospital, Athens, Greece

## Abstract

**Introduction:**

As a result of the notable progress in HIV / AIDS prevention and treatment globally, the HIV epidemic is considered controlled to the extent that individuals living with HIV manage to have a similar life expectancy to HIV-negative individuals and a good level of health. However, the “epidemic” related to the stigma of HIV-positive individuals, particularly of Men who have Sex with Men (MSM), still remains an issue, while it has been associated with a profound negative impact on individuals’ mental health.

**Objectives:**

The present study aimed to compare anxiety, depression, social support, loneliness, and psychological resilience between MSM with and without HIV. Furthermore, the study investigated the correlates of HIV related stigma in MSM with HIV.

**Methods:**

The sample of the study comprised a total of 139 adult MSM. According to their self-report 84 individuals were HIV-negative (age in years: M = 24.58, SD = 5.55), while the remaining 55 individuals were HIV-positive (age in years: M = 38.99, SD = 10.95). Furthermore, the majority of individuals in both groups reported Greek nationality. Participants completed a questionnaire on socio-demographic characteristics, the Hospital Anxiety and Depression Scale to assess anxiety and depression, the UCLA Loneliness Scale to measure loneliness, the Brief Resilience Scale (BRS) to evaluate psychological resilience, and the Multidimensional Scale of Perceived Social Support (MSPSS) to assess social support. HIV-positive individuals additionally completed the HIV Stigma Scale-Brief Version, a brief scale measuring social stigma related to HIV.

**Results:**

According to the findings, MSM with and without HIV did not differ in anxiety (*p* = 0.908), depression (*p* = 0.904), social support (*p* = 0.657), loneliness (*p* = 0.086), and psychological resilience (*p* = 0.600). Furthermore, it emerged that among HIV-positive MSM, stigma was positively associated with anxiety (*r* = 0.479, *p* < 0.001), depression (*r* = 0.479, *p* < 0.001), and loneliness (*r* = 0.532, *p* = 0.001), while the correlation was negative with social support (*r* = -0.456, *p* < 0.001) and resilience (*r* = -0.400, *p* < 0.003). No significant association was found between stigma and age in HIV-positive individuals (*r* = 0.099, *p* = 0.474).

**Conclusions:**

In conclusion, the findings of this study identified possible risk factors as well as protective factors for the mental health of HIV-positive MSM. As long as stigma associated with HIV continues to be a risk factor for the development of psychosocial problems in those living with HIV, it is advisable for both policymakers and the research community to take more proactive steps in order to offer the necessary support and attention to those who are living with HIV and experiencing multiple forms of stigma.

**Disclosure of Interest:**

None Declared

